# Genetic dissection of soybean lodging tolerance in recombinant inbred-line populations of major Japanese and modern US varieties

**DOI:** 10.1270/jsbbs.24088

**Published:** 2025-06-21

**Authors:** Ai Hishinuma, Atsunori Fukuda, Takuma Sugimoto, Osamu Uchikawa, Shigeki Morita, Ryohei Okuno, Shin Kato, Akio Kikuchi, Takashi Sayama, Yuko Yokota, Takehiko Shimizu, Fumio Taguchi-Shiobara, Eri Ogiso-Tanaka, Akito Kaga, Kaori Hirata, Tetsuya Yamada, Kenichiro Fujii, Feng Li, Makita Hajika, Masao Ishimoto

**Affiliations:** 1 Tohoku Agricultural Research Center (TARC), National Agriculture and Food Research Organization (NARO), 297 Uenodai, Kariwano, Daisen, Akita 019-2112, Japan; 2 Institute of Crop Science (NICS), NARO, 2-1-2 Kannondai, Tsukuba, Ibaraki 305-8518, Japan; 3 Hyogo Agricultural Institute for Agriculture, Forestry and Fisheries, 1533 Minamino-oka, Befu, Kasai, Hyogo 679-0198, Japan; 4 Fukuoka Prefectural Government Office, 7-7 Higashikoen, Hakata-ku, Fukuoka City, Fukuoka 812-8577, Japan

**Keywords:** soybean, lodging tolerance, mechanical suitability, quantitative trait loci, marker-assisted selection

## Abstract

In soybean production, lodging poses a significant challenge to modern mechanized agriculture, such as the use of combine harvesters. Most Japanese varieties are prone to lodging because of the local weather conditions, such as wind and rain, resulting in a decline in productivity. In the United States (US), where mechanized agricultural production systems are prevalent, lodging tolerance (LT) is essential in soybean breeding. We thus used two recombinant inbred-line (RIL) populations developed by crossing major Japanese and modern US varieties for the genetic dissection of LT. One reliable quantitative trait locus (QTL) for lodging angle, *qLT13-1*, was identified from the first RIL population under two experimental conditions, early and late maturity groups of the first RILs in Ibaraki in 2018, and it accounted for 20.7%–20.9% of the phenotypic variation. An allele at *qLT13-1* from a US variety was effective in improving LT under most experimental conditions. In addition, a QTL for LT was valid in the same genetic region of the other RIL populations. The effective allele, *qLT13-1* is thus expected to be important for improving LT in soybean breeding, particularly in Japanese varieties.

## Introduction

Global soybean production is growing continuously because of increasing demand. This growth is realized through an increase in both cultivated area and yield per unit area (Ainsworth *et al.* 2012, Agricultural Market Information System (https://www.amis-outlook.org/)). At the same time, soybean yields in Japan have not only failed to increase but even decreased gradually over the past 20 years (Chen 2018). During this period, the scale of cultivation has significantly expanded in Japan, and it is predicted that this expansion will continue because of further decreases in the number of farming households and agricultural workers (Kobayashi and Kunimitsu 2016); however, the performance of Japanese varieties has not caught up with this rapid expansion. When the cultivation scale expands, the use of large agricultural machinery, such as strip-till machines and combine harvesters, becomes indispensable for cultivation management. Depending on the characteristics of the varieties, mechanized farming can significantly increase cultivation and harvest losses. Most Japanese varieties are susceptible to pod shattering, which can increase harvest losses under mechanical harvesting (Tsuchiya 1987, Yamada *et al.* 2017). Modern United States (US) varieties are predominantly resistant to pod shattering, and recently, the responsible gene pod dehiscence 1 (*pdh1*) was identified and successfully incorporated into Japanese varieties to reduce losses caused by mechanical harvesting (Funatsuki *et al.* 2012, 2014, Yamada *et al.* 2013, 2017).

Apart from pod shattering, lodging is another factor that negatively impacts cultivation management and harvest operations. When lodging occurs, mechanical management operations can cause damage to plants and lead to yield losses (Palomeque *et al.* 2010, Philbrook and Oplinger 1989). Furthermore, lodging during the growing stages reduces productivity owing to light interception with the canopy (Board 2001, Cooper 1971, Shaw and Weber 1967). There have been several reports of quantitative trait loci (QTL) for lodging tolerance (LT) in soybean, but most of these detected QTL are located near the *Dt1* and *E3*, which are involved in growth habit and flowering time (Hwang and Lee 2019, Lee *et al.* 1996, Mansur *et al.* 1993, Orf *et al.* 1999). Since these traits are important factors that also affect soybean cultivation methods and suitable growing regions, it is difficult to widely utilize these QTL for LT breeding. Most soybeans in Japan are produced in converted paddy fields, and Japan experiences frequent rain and is often affected by strong winds, such as typhoons, which often cause lodging of soybean plants (Igita *et al.* 1984, Rehman *et al.* 2024, Saitoh *et al.* 2012). Owing to these environmental conditions, it is difficult to control the occurrence of lodging and the evaluation of LT. As a result, despite the fact that Japan has an environment that is prone to lodging, no significant progress has been made in LT breeding for Japanese soybean varieties. Therefore, at present, lodging remains one of the major causes of low yields and production instability in soybean cultivation in Japan. Conversely, the higher yields observed in major soybean-producing countries, including the US, Canada, Brazil, China, and Argentina can be attributed to continuous breeding, with one contributing factor being the improvement of LT (de Felipe *et al.* 2016, Jin *et al.* 2010, Morrison *et al.* 2000, Rogers *et al.* 2015, Umburanas *et al.* 2022, Wilcox 2001, Wu *et al.* 2015). Therefore, varieties from these countries may carry genetic factors that enhance LT without negatively affecting yield, and these factors could potentially be utilized to improve LT in Japanese soybean varieties.

In this study, we conducted a genetic analysis of LT using two recombinant inbred-line (RIL) populations derived from crosses between major Japanese varieties and modern US varieties and identified a reliable QTL on soybean chromosome (Chr.) 13. In addition, we selected recombinant residual lines (RHLs) from RILs using the nearest marker to the major QTL to validate their effects on LT, seed yield, and other agronomic traits. This is the first study to identify a LT-related QTL in modern US soybean varieties and evaluate its efficacy in breeding of lodging-tolerant varieties in Japan.

## Materials and Methods

### Plant materials and field tests

**Fig. 1.**
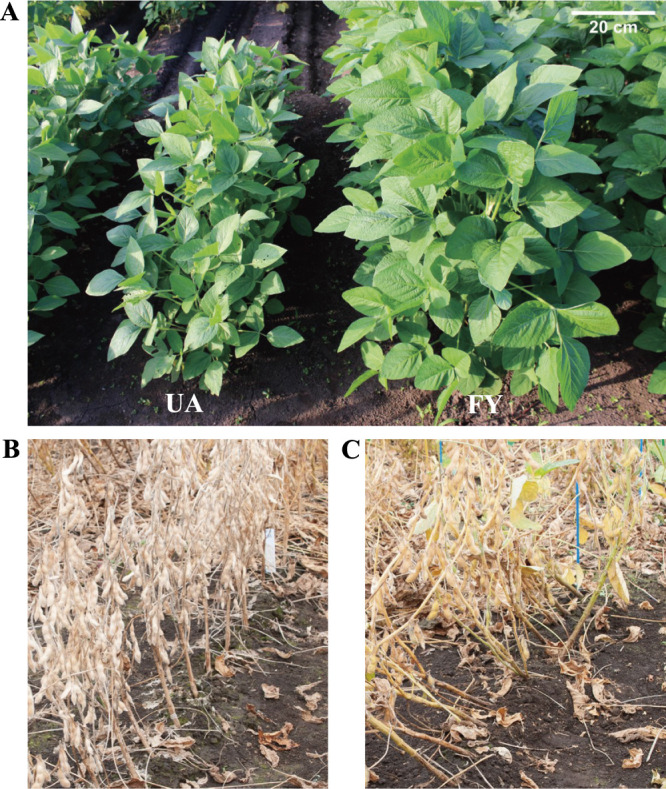
Representative images of Fukuyutaka (FY) and UA 4805 (UA) plants in the field. (A) UA and FY plants sown on May 30, 2017 in Akita (images taken on August 4, 2017). (B, C) UA and FY, respectively, sown on June 24, 2016 in Ibaraki (images taken on October 30, 2016).

Two major Japanese varieties, ‘Fukuyutaka’ (FY) and ‘Sachiyutaka’ (SY), and two modern US varieties, ‘UA4805’ (UA) and ‘LD00-3309’ (LD), which markedly differ in terms of LT as indicated by their Lodging scores (Table 1, Fig. 1), were used. FY is a determinate cultivar developed with seed quality suitable for tofu production at the Kyushu National Agricultural Experiment Station (current Kyushu Okinawa Agricultural Center, NARO: KARC/NARO) in 1980 (Ohba *et al.* 1982), and it is currently the most widely cultivated variety in Japan. SY is also a determinate cultivar developed for tofu production from a backcross generation of a cross between FY and ‘Enrei’ (ER) using ER as the recurrent parent at KARC/NARO (Takahashi *et al.* 2004). UA is a determinate cultivar released by the Arkansas Agricultural Experiment Station in 2005 and has a high yield potential and LT (Chen *et al.* 2006). LD is an indeterminate cultivar released by the Illinois Agricultural Experiment Station in 2006 and has a high yield potential (Diers *et al.* 2006). A combination of the five flowering-related loci and one growth habit locus of the four varieties, *E1*, *E2*, *E3*, *E4*, *Tof11*, and *Dt1* (Bernard 1971, 1972, Buzzell 1971, Buzzell and Voldeng 1980, Lu *et al.* 2020), is shown in Table 1.

An FU-RIL population (367 lines) was developed using the single-seed-descent method from an FY × UA cross from the F_2_ population to the F_6_ generation. FY and UA had identical allelic combinations at four major flowering-related loci, except for *Tof11*, and one growth habit locus. An SL-RIL population (328 lines) was developed using the single-seed-descent method from an SY × LD cross from the F_2_ population to the F_7_ generation. SY and LD had different allelic combinations at three major flowering-related loci, *E1*, *E3*, and *Tof11*, and one growth habit-related locus, *Dt1* (Table 1).

Field tests for FU-RILs and their parental varieties were conducted in four different locations in Japan, namely, Akita (39°32ʹ N, 140°22ʹ E), Ibaraki (36°01ʹ N, 140°06ʹ E), Fukuoka (33°30ʹ N, 130°34ʹ E), and Hyogo (34°55ʹ N, 134°53ʹ E), in 2017 and 2018 (Supplemental Fig. 1). A key difference among these locations is latitude, which influences day length and light intensity (factors that significantly affect plant growth). Each plot was randomized, with one (Akita, Ibaraki, and Fukuoka) or two (Hyogo) replicates per year. In Ibaraki, two FU-RILs experimental groups were considered, i.e., the early maturity group (EMG) sown in early June and the late maturity group (LMG) sown in early July. Field tests for SL-RILs and their parental varieties were conducted in Akita from 2019 to 2022. Each plot was also randomized, with no replicate per year. To estimate the effect of the QTL on different FU-RILs growth habits, indeterminate growth habits were selected for SL-RILs, and the *e1*/*E3* combination of flowering-related loci was chosen for proper cultivation in Akita. Further, the *Tof11* locus was selected for 34 SY-type lines and 26 LD-type lines, representing approximately half of the tested lines. The experimental conditions and respective test names are listed in Table 2.

### Evaluation of LT and other agronomical traits in RILs and the parents

In the FU-RILs, LT was evaluated in five to twelve consecutive plants in the central row as the inclination angle of the main stems, ranging from 0° (no lodging) to 90° (completely lodged), using a digital bevel meter (Niigata Seiki Co., Ltd., Niigata, Japan) at the time of maturity. The number of days to flowering (FT) was recorded when more than 50% of plants in the plots flowered. Other agronomic traits, including main stem length and seed weight, were measured in five to twelve plants in the same central row where LT was measured. In SL-RIL population, LT was visually recorded on a scale from 0 (no lodging) to 5 (completely lodged) at maturity. Similar to the method used for the FU-RIL population, other agronomic traits were measured for three to six plants.

### Calculation of broad-sense heritability for LT

The environmental variance, genetic variance, and broad-sense heritability of the lodging angle and main stem length were calculated using the data of the parents and FU-RILs of I17-FUE, I18-FUE, I17-FUL, and I18-FUL (Table 2).

### Molecular marker analysis and linkage mapping

Total DNA of FU-RILs and SL-RILs was extracted from leaflets or cotyledonary tissue using an automatic DNA isolation system (BioSprint 96 DNA Plant Kit, Qiagen, Hilden, Germany) and according to the procedure described by Khosla *et al.* (1999). In FU-RILs, F_6_ plants were genotyped using the whole-genome simple sequence repeat (SSR) marker panel (WGSP) ver. 2 (Fujii *et al.* 2018, Sayama *et al.* 2011). In addition to the markers in the panel, the polymorphic SSR markers, BARCSOYSSR_13_1538, BARCSOYSSR_13_1670, and BARCSOYSSR_13_1804 (Song *et al.* 2010)—were genotyped. These markers were located near a major QTL, *qLT13-1*, and were used to estimate the region containing *qLT13-1*. A linkage map was constructed using 152 polymorphic markers. Using these markers, linkage analysis was performed with MAPMAKER/EXP 3.0b software (Lander *et al.* 1987), and genetic distance was calculated with Kosambi’s mapping function (Kosambi 1943). The marker order with the highest likelihood within Chr. 13 was adopted. Genotypes of the *E1*, *E2*, *E3*, and *E4* loci were determined using the nearest SSR markers as described by Tsubokura *et al.* (2014). The genotype of *Tof11* was determined using a genetic marker that detects differences in single-nucleotide deletions (Lu *et al.* 2020). In the SL-RILs, the genotypes of the *E1*, *E3*, and *Tof11* loci of F_6_ plants were determined as in the FU-RILs. The genotype of *Dt1* was determined using the SSR marker Sat_286 located near *Dt1* (Kato *et al.* 2018). Sixty of the 328 SL-RILs with genotypes *e1*/*E3*/*Tof11*/*Dt1* and *e1*/*E3*/*tof11*/*Dt1* were used for LT evaluation. Furthermore, to investigate the association between the genotypes around *qLT13-1* and the lodging scores in the SL-RILs, the genotypes of six SSR markers, BARCSOYSSR_13_1452, BARCSOYSSR_13_1500, BARCSOYSSR_13_1545, BARCSOYSSR_13_1580, BARCSOYSSR_13_1596, and BARCSOYSSR_13_1670, were determined.

### QTL analysis

In FU-RILs, QTL analysis was performed through the composite interval mapping method with Windows QTL Cartographer V 2.5 software (https://statgen.ncsu.edu/qtlcart/WQTLCart.htm). The genome was scanned at 1-cM intervals, and the additive effects and logarithm of odds (LOD) were estimated. A permutation test was conducted 1,000 times for each trait and experiment, and the threshold value of the LOD peak was set at *p* = 0.05. To improve the normality and homogeneity of variance, the lodging angle data were log-transformed for QTL analysis.

### Evaluation of the QTL effect in residual heterozygous lines from the RILs

To evaluate the effects of the QTL on Chr. 13, we selected residual heterozygous lines (RHLs), in which the genomic region of interest was heterozygous and the other regions were homozygous (Tuinstra *et al.* 1997, Yamanaka *et al.* 2005). Four RHLs (termed RHL-026, RHL-185, RHL-186, and RHL-277) were selected from the FU-RILs (F_6_). RHL-185 and RHL-186 were selected from the EMG with the UA allele at *Tof11*, and RHL-026 and RHL-277 were selected from the LMG with the FY allele at *Tof11*. The progeny of the heterozygous plants was genotyped using several SSR markers to narrow down the region of the QTL, including BARCSOYSSR_13_1538, which was the nearest to the QTL (Supplemental Table 1, BARC-SOYSSR_1.0; Song *et al.* 2010). DNA was extracted from F_6_ seeds and genotyped using the selected markers. These lines were F_7_ in 2018, F_8_ in 2019, and F_10_ in 2021. These lines were planted in Ibaraki to validate QTL effects. The field experiment was conducted as in the FU-RILs. The experimental conditions for the RHLs are listed in Supplemental Table 2.

### Confirmation of the insertion of a retrotransposon at *PH13* on Chr. 13

The insertion of a retrotransposon at *PH13* on Chr. 13 (Qin *et al.* 2023) was confirmed using molecular markers specifically designed for PCR detection (Supplemental Table 1). The presence of fragment insertions was confirmed using agarose gel electrophoresis. Primer sequences used for this analysis, with the exception of WGSP ver. 2, are shown in Supplemental Table 1.

## Results

### Evaluation of LT and related traits of Japanese and US varieties

The two US varieties showed better LT than the Japanese varieties (Table 1, Fig. 1). To assess stability across environments, LT of FU and UA was investigated at four locations over 1 to 2 years. UA showed a significantly smaller lodging angle than FY under seven environmental conditions. However, there were no significant differences between two environmental conditions, A17-FUE and F18-FUL (Supplemental Fig. 2). We also observed an earlier FT and significantly shorter main stem length for UA than for FY (Table 1). Because flowering-related loci influence main stem length and main stem length affects LT (Lin and Nelson 1988, Wilcox and Sediyama 1981), the FU-RIL population was divided into two groups based on the genotype combinations of the flowering-related loci. One of the parental varieties of SL-RILs, LD, showed a significantly lower lodging score than SY, another parental cultivar (Table 1). Additionally, compared to SY, LD had an earlier flowering time and significantly longer main stem length (Table 1). For the SL-RILs, the analysis population was designed as described in “Materials and Methods,” considering flowering-related loci, which influence main stem length.

### Evaluation of the effect of *Tof11* in FU-RILs

All lines of the FU-RIL population were grown in I16-FU for genotyping and seed multiplication with lodging prevention measures. The FT and main stem length were recorded simultaneously. Because FY and UA have different alleles at the *Tof11* locus, a 1:1 ratio was expected in FU-RILs. The number of lines segregated 164:191 by *Tof11* alleles, which is consistent with the expected segregation ratio (χ^2^ = 2.38, 0.10 < *p* < 0.20). The distribution of FT in the FU-RIL population ranged from 44 to 56 days, with an average of 49 days, and two gradual peaks were observed (Supplemental Fig. 3A). These peaks were separated by the *Tof11* genotype, with an average FT of 51 and 48 days for the *Tof11* and *tof11* groups, respectively (Supplemental Fig. 3A). The broad distribution of the FT when divided by *Tof11* genotype suggests that there may be an effect of loci other than the flowering related loci considered in this study. The difference in FT by *Tof11* genotype also had a significant effect on main stem length, with average main stem lengths of 65.4 cm for the *Tof11* group and 55.0 cm for the *tof11* group (Supplemental Fig. 3B), showing a significant correlation between FT and main stem length (Supplemental Fig. 4). Therefore, in subsequent experiments, the FU-RIL population was divided into two groups, the EMG and the LMG, according to the *Tof11* genotype.

### QTL analysis for lodging angles in FU-RILs

FU-RILs were grown, and lodging angle data were obtained from four locations (Table 2). One maturity group, the EMG of the FU-RIL population, was evaluated in Akita and Ibaraki in 2017 and 2018, and the other maturity group, the LMG of the FU-RIL population, was evaluated in Ibaraki, Hyogo, and Fukuoka in 2017 and/or 2018. The frequency distribution of lodging angles from cultivation at each location is shown in Supplemental Fig. 5. Broad-sense heritability ranged from 0.65 to 0.92 in Ibaraki, where all lodging angle data showed significant positive pairwise correlations with other environments, suggesting that lodging angles had relatively high heritability (Supplemental Table 3). For QTL analysis, lodging angle data were used only if they showed positive pairwise correlations with other environments to identify stable QTLs; therefore, A17-FUE and F18-FUL were excluded (Supplemental Table 4).

**Fig. 2.**
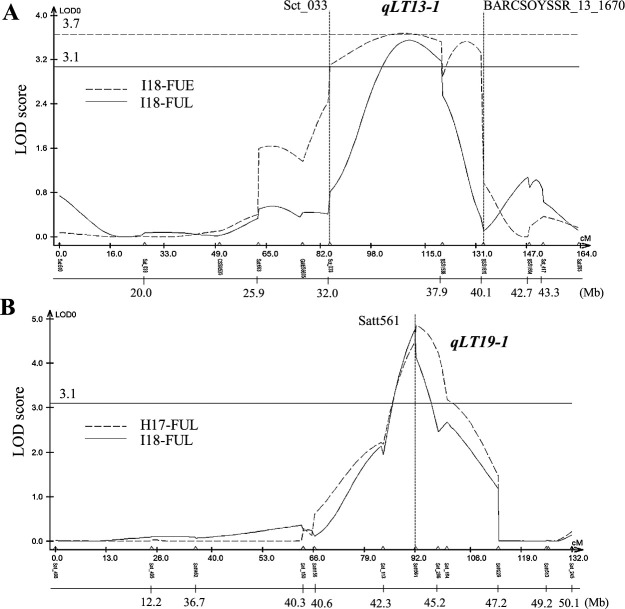
LOD plots for QTLs associated with lodging angle in FU-RILs. Lodging angle data were logarithmically transformed. “BSS” represents “BARCSOYSSR_13_.” (A) *qLT13-1* on Chr. 13. The LOD threshold values at a 5% probability level were 3.1 and 3.7 in I18-FUE and I18-FUL, respectively. (B) *qLT19-1* on Chr. 19. The LOD threshold value at a 5% probability level was 3.1. Only LOD plots for significantly detected QTLs are shown.

Among the parental varieties FY and UA of FU-RILs, polymorphisms were observed in 149 of 181 analyzed markers, from a total of 352 SSR markers, as assessed with the WGSP ver. 2 (Fujii *et al.* 2018, Sayama *et al.* 2011). A preliminary QTL analysis suggested the presence of a QTL on Chr. 13, so additional SSR markers, BARCSOYSSR_13_1538, BARCSOYSSR_13_1670, and BARCSOYSSR_13_1804 (Song *et al.* 2010), were added for the construction of a linkage map, covering 2451.7 cM across 20 molecular linkage groups, with an average marker interval was 21.1 cM. In the QTL analysis of lodging angle using this linkage map, two QTLs were detected across multiple environments: *qLT13-1* on Chr. 13 (linkage group F: LG-F), and *qLT19-1* on Chr. 19 (LG-L), respectively (Table 3, Fig. 2). The *qLT13-1* locus, detected between Sct_033 (85.12 cM) and BARCSOYSSR_13_1670 (133.4 cM) in two, I18-FUE and I18-FUL, out of seven environments, had LOD scores of 3.7 and 3.6, respectively, with the LOD score peak located near BARCSOYSSR_13_1538. The additive effect of the FY allele was positive, indicating that the UA allele at *qLT13-1* is associated with strong LT (Table 3). In contrast, the QTL on Chr. 19, *qLT19-1* detected in two, H17-FUL and I18-FUL, out of seven environments, had LOD scores of 4.9 and 4.8, respectively, and its LOD score peak was located near Satt561. Since the additive effect of the FY allele was negative, *qLT19-1* was associated with strong LT when the FY allele was present (Table 3). As UA had better LT than FY, we focused on *qLT13-1*, which was expected to improve LT with the UA allele.

### Effect of *qLT13-1* on LT under different environmental conditions

**Fig. 3.**
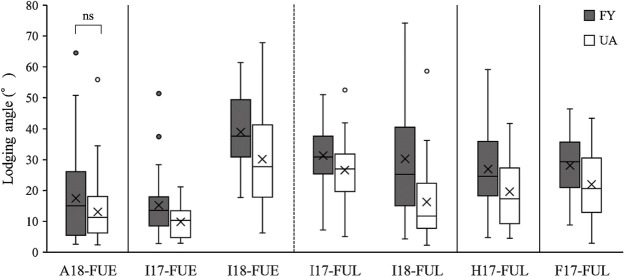
Comparison of lodging angles associated with the *qLT13-1* allele (BARCSOYSSR_13_1538) in FU-RILs. The shaded and white boxes indicate FY and UA alleles, respectively. Significant differences (*p* < 0.05) were detected by performing Welch’s *t*-test using log-transformed lodging angles except for pairs designated ns (not significant).

**Fig. 4.**
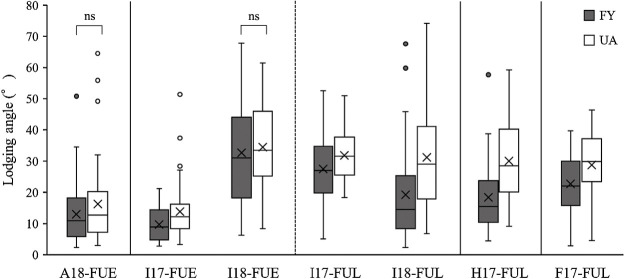
Comparison of lodging angles associated with the *qLT19-1* allele (Satt561) in FU-RILs. The shaded and white boxes indicate FY and UA alleles, respectively. Significant differences (*p* < 0.05) were detected by performing Welch’s *t*-test using log-transformed lodging angle except for pairs designated ns (not significant).

A significant QTL, *qLT13-1*, was detected in only two of the seven environments investigated. Using the *qLT13-1* adjacent DNA marker, BARCSOYSSR_13_1538, we assessed the effects of LT on the chromosomal regions of *qLT13-1* in the seven environments that were used for the QTL analysis. In UA, the *qLT13-1* allele significantly enhanced LT in almost all the environments, except for A18-FUE (Fig. 3, Table 4). However, it had no adverse effects on seed yield or 100-seed weight (Table 4). Further, owing to the *qLT13-1* allele, length of the main stem of the plants decreased even though there was no shortening in FT. Additionally, there was no considerable decrease in number of main stem nodes. Since, the main stem length was significantly shortened, i.e., in UA, the *qLT13-1* allele significantly shortened the main stem internode length. The effect of *qLT19-1*, the QTL associated with a strong LT for the FY allele, was also employed to assess the effects of LT on chromosomal regions using the adjacent DNA marker, Satt561. The *qLT19-1* allele in FY significantly enhanced LT in almost all environments, except for A18-FUE and I18-FUE (Fig. 4, Table 5). Further, its effects on flowering time and the shortening of the main stem length of the plants were negligible. There was no significant effect on the number of main stem nodes (Table 5). These observations indicated that *qLT13-1* and *qLT19-1* are effective QTLs that enhance LT without adverse effect on yield.

### Narrow down the effective region of *qLT13-1*

The region of *qLT13-1* is broad, spanning 8.2 Mbp between the Sct_033 and BARCSOYSSR_13_1670 markers (Fig. 2); therefore, we increased the number of DNA markers in this region to narrow it down. We designed and selected 14 SSR markers according to WGSP ver. 2 (Fujii *et al.* 2018, Sayama *et al.* 2011). We selected four RIL lines, FU-026, 185, 186, and 277, which were heterozygous in the region of *qLT13-1*, and developed four pairs of recombinant fixed lines originating from these RHLs (Supplemental Table 5). Although the results for the four pairs were not stable across years, a minimum region of about 6.6 Mbp from BARCSOYSSR_13_1476 (36.7 Mbp) to WGSP13_0160 (43.3 Mbp) seemed necessary. These results suggest that a relatively broad region of UA was required for LT.

### Evaluation of the effect of QTL for LT in SL-RILs

**Fig. 5.**
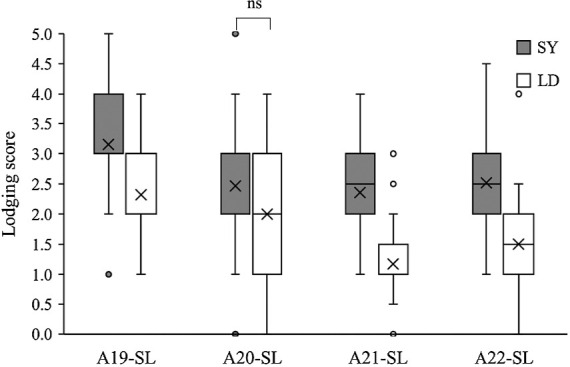
Comparisons of lodging scores associated with *qLT13-1* alleles (BARCSOYSSR_13_1545) in SL-RILs over a period of 4 years. The shaded and white boxes indicate the SY and LD alleles, respectively. Significant differences (Welch’s *t*-test; *p* < 0.05) were observed in all the experiments expect for experiment A20-SL.

Apart from UA, the other US cultivar LD also has better LT than Japanese varieties (Table 1). We developed an SL-RIL population and selected 60 lines with *e1*/*E3* flowering-related loci and an indeterminate growth habit suitable for cultivation in Akita (Supplemental Fig. 6). The *Tof11* locus was randomly selected for analysis in 34 SY-type lines and 26 LD-type lines, which together represent approximately half of the tested lines. To evaluate the effect of LT in SL-RILs, we conducted a field experiment over a period of 4 years. Using the *qLT13-1* adjacent DNA marker, BARCSOYSSR_13_1545, we assessed the effects of LT in this chromosomal region on Chr. 13 in four environments (Fig. 5, Table 6). The LD allele significantly improved LT in almost all the environments, except in A20-SL. It also significantly shortened main stem length, while minimally affecting the number of main stem nodes, i.e., both the LD and UA alleles shortened the main stem internode length of the plants. Additionally, they exerted no effect on FT, 100-seed weight, and seed yield (Table 5). These observations suggest that the QTL, *qLT13-1*, present in the two US varieties, UA and LD, effectively contribute to LT.

### Analysis of neighboring genes of *qLT13-1*

In both RIL populations, FU-RILs and SL-RILs, the region of *qLT13-1* shortens the main stem length but it does not reduce the number of main stem nodes, i.e., it may shorten an internode length of the main stem (Tables 4, 6). Three genes related to main stem length have been reported in this region, i.e., *GA2ox8A*, *GA2ox8B*, and *PH13* (Qin *et al.* 2023, Wang *et al.* 2021), and QTLs for main stem length were detected with FU-RILs in the vicinity of *PH13* (Supplemental Fig. 7). *PH13* is involved in the regulation of plant height by the insertion of a *Ty1/Copia*-like retrotransposon (Qin *et al.* 2023). We then confirmed the insertion of a retrotransposon in *PH13* for the four varieties, UA, LD, FY, and SY, which are the parents of the two RIL populations used in this study. UA and LD harbored a retrotransposon in *PH13*, while FY and SY did not (Supplemental Fig. 8). This suggests that these two US varieties possess a *PH13* allele that reduces plant height.

## Discussion

LT is an essential trait in modern mechanized agriculture (Weber and Fehr 1966). In soybean cultivation, combine-harvesting losses due to lodging are estimated at approximately 20% (Uchikawa *et al.* 2006). Furthermore, lodging during growth stages also reduces productivity owing to light interception within the canopy (Board 2001, Cooper 1971, Shaw and Weber 1967). However, lodging, is an unstable and environmentally dependent trait (Igita *et al.* 1984), that complicates selection for tolerance through breeding. There are several reports of QTL analyses for LT in soybeans, with many of the detected QTLs located near the *Dt1* or *E3* locus on Chr. 19, which are related to growth habit and flowering time (Hwang and Lee 2019, Lee *et al.* 1996, Mansur *et al.* 1993, Orf *et al.* 1999). These genes, including other flowering-related loci such as *E1*, *E2*, *E4*, and *Tof11* determine the growth period and plant size based on day length at the cultivation site (Cober and Morrison 2010, Kato *et al.* 2018, Lee *et al.* 1996, Lu *et al.* 2020, Mansur *et al.* 1993). Although LT is a labile trait, we observed a high LT heritability in this study (Supplemental Table 3), possibly owing to the stable lodging evaluation method employed, which involves the use of a digital bevel meter. The relatively high heritability of LT, even though lower than that of main stem length (Supplemental Table 3), suggesting that LT, while influenced by environmental factors, is primarily controlled by genes. Further, despite its instability, LT has been steadily improved through breeding in the US and other countries. First, we compared LT of the US and Japanese varieties. The US varieties were clearly superior in terms of LT (Table 1, Fig. 1), thus we developed RILs from the progeny of crosses between the US and Japanese varieties and performed genetic analyses of LT.

We investigated the genotypes of the flowering-related loci and selected the first combination, FY and UA, which shared four of the five major flowering-related loci (Table 1). The resulting FU-RIL population (367 lines) was divided into two groups, EMG (harboring *tof11*) and LMG (harboring *Tof11*), based on the genotype of *Tof11*, and was used for subsequent QTL analysis (Supplemental Fig. 3). In the second combination, SY and LD, the parental varieties differed in three of the five flowering-related loci (Table 1). We selected 60 lines with *e1/E3/Tof11* or *e1/E3/tof11* flowering-related loci and an indeterminate growth habit (*Dt1*) for field experiments in Akita. These adjustments of flowering-related loci led to the successful confirmation of the effect of QTL for improving LT. That is, a comparison of genotypes using DNA markers adjacent to QTLs in multi-year trials in Akita showed a significant difference in lodging, indicating that *qLT13-1* is involved in improving LT in LD as well. However, it is not clear whether the genes responsible for LT in this effective QTL region are the same. Further analysis of the QTL region using several US varieties is necessary. In the FU-RILs, two QTLs, *qLT13-1* on Chr. 13 and *qLT19-1* on Chr. 19, were detected from two respective trials out of the seven environments (Table 3, Fig. 2). *qLT13-1* and *qLT19-1* had greater effects on LT in the UA and FY alleles, respectively. Since *qLT19-1* is located near the *E3* gene (Watanabe *et al.* 2009) but had little effect on FT (Table 5), it is necessary to further investigate whether there are differences in the vicinity of the *E3* sequence between the parents and also clarify the relationship between *qLT19-1* and *E3*.

*qLT13-1* was positioned in a region of approximately 6.6 Mbp between BARCSOYSSR_13_1476 and WGSP13_0160. Recently, a major QTL for trailing growth in wild soybeans was isolated from this region, which included two gibberellin 2-oxidase 8 genes (*GA2ox8A* and *GA2ox8B*) (Wang *et al.* 2021). This QTL played an important role during domestication, as increased copy numbers of *GA2ox8A* and *GA2ox8B* reduced trailing growth and shoot length. The main stem length decreased in relation to the copy number of the two *GA2ox8* genes, and the reduction in the main stem length was primarily due to a significant decrease in internode length. In the present study, *qLT13-1* shortened the main stem length without reducing the number of main stem nodes (Table 4), suggesting that differences in the copy number or expression of the two *GA2ox8* genes could contribute to improved LT. Long-read sequencing is needed to confirm the precise copy number of the two *GA2ox8* genes in the two US varieties, UA and LD, compared with the two Japanese varieties, FY and SY. More recently, another gene regulating main stem length (*PH13*) was isolated from the *qLT13-1* region (Qin *et al.* 2023) (Supplemental Fig. 7). *PH13* encodes a WD40 protein, and the insertion of a retrotransposon into this gene leads to a truncated *PH13* protein with reduced interaction with *GmCOP1s*, resulting in reduced plant height. *PH13* is involved in the response to low blue light and inhibits the excessive stem elongation syndrome at high latitudes and under high-density planting. We confirmed the insertion of a retrotransposon in *PH13* in the two US varieties, whereas no such insertion was found in the two Japanese varieties (Supplemental Fig. 8). As a broad region of *qLT13-1*, including the *PH13* and *GA2ox8* genes, affects LT, it is possible that all these genes are necessary or that other unknown genes may also be involved in LT. Further research is required to identify the causative gene(s) responsible for LT within the *qLT13-1* region.

The presence of the *qLT13-1* region in UA and LD had no adverse effects on seed yield or 100-seed weight (Tables 4, 6). These results suggest that this QTL may be practically effective for breeding without having a negative impact on the yield-related traits. Even though, we successfully identified *qLT13-1* as a QTL for conferring improved LT in the modern US varieties, further evaluation using combined harvesting is needed to confirm the loss reduction effect of this QTL given that in this study, we determined seed yield following hand-harvesting. In light of this study, it is important to promptly utilize this QTL to improve LT in Japanese varieties suitable for mechanical management operations.

## Author Contribution Statement

MH and MI designed and directed the study. AH, SK, and AK in Akita and AF, Ibaraki, TS, Hyogo, OU, SM, and RO in Fukuoka conducted field experiments and obtained data in each environment. AH and AF performed QTL analyses. TS and AF developed FU-RILs and RHLs. FT, TS, AF, EO, AK, YY, and TS constructed the genetic linkage map and performed DNA analysis. AK, KH, TY, and KF developed the SL-RILs and AK and KF performed DNA analysis. FL performed genotyping of *PH13*. AH organized the overall data and performed statistical analysis; AH and AF drafted the manuscript. MI devised test materials and provided instruction in writing.

## Supplementary Material

Supplemental Figures

Supplemental Tables

## Figures and Tables

**Table 1.** Comparison of the agronomic traits of the four cultivars used in this study

Cultivar		Lodging score*^a^*	Main stem length (cm)	Days to flowering*^b^*	Genotypes of flowering related loci					Genotype of growth habit
					*E1*	*E2*	*E3*	*E4*	*Tof11*	
UA4805	(UA)	0.8	50.0	48	*E1*	*E2*	*E3*	*E4*	*tof11*	*dt1*
Fukuyutaka	(FY)	3.1	87.9	52	*E1*	*E2*	*E3*	*E4*	*Tof11*	*dt1*
		***	***	–						
LD00-3309	(LD)	0.0	65.1	30	*e1*	*E2*	*E3*	*E4*	*tof11*	*Dt1*
Sachiyutaka	(SY)	1.8	45.3	39	*E1*	*E2*	*e3*	*E4*	*Tof11*	*dt1*
		***	***	–						

Data were obtained in Ibaraki in 2016. *^a^* Lodging score: 0 (no lodging) to 4 (completely lodged). *^b^* Days to flowering determined for each plot. No statistical analysis was performed. Data on LD and SY were obtained in 2017. *** significantly different value at *p* < 0.001 based on Welch’s *t*-test.

**Table 2.** Experimental conditions and test name at four locations

RIL type	Location	Test name	Year	Number of lines	*Tof11* allele	Group*^a^*	Planting Date	Plant separation distance (m)	Row spacing (m)	Field type	Fertilization (kg ha^–1^)			
											N	P_2_O_5_	K_2_O	MgO
FU-RILs	Akita	A17-FUE	2017	99	*tof11*	EMG	30-May	0.12	0.75	Upland field	24	160	80	136
		A18-FUE	2018	99	*tof11*	EMG	25-May	0.12	0.75	Upland field	24	160	80	136
	Ibaraki	I16-FU	2016	367	Both	Both	24-Jun	0.20	0.80	Upland field	18	280	60	100
		I17-FUE	2017	91	*tof11*	EMG	8-Jun	0.10	0.70	Upland field	30	500	100	300
		I18-FUE	2018	91	*tof11*	EMG	7-Jun	0.10	0.70	Upland field	30	500	100	300
		I17-FUL	2017	81	*Tof11*	LMG	3-Jul	0.10	0.70	Upland field	30	500	100	300
		I18-FUL	2018	81	*Tof11*	LMG	3-Jul	0.10	0.70	Upland field	30	500	100	300
	Hyogo	H17-FUL	2017	81	*Tof11*	LMG	19-Jun	0.10	0.70	Upland field	40	120	160	Non
	Fukuoka	F17-FUL	2017	74	*Tof11*	LMG	8-Jun	0.15	0.70	Paddy field	Non	Non	Non	Non
		F18-FUL	2018	74	*Tof11*	LMG	7-Jun	0.15	0.70	Paddy field	Non	Non	Non	Non
SL-RILs	Akita	A19-SL	2019	60	*tof11*	–	27-May	0.12	0.75	Upland field	24	160	80	136
		A20-SL	2020	60	*tof11*	–	29-May	0.12	0.75	Upland field	24	160	80	136
		A21-SL	2021	60	*tof11*	–	1-Jun	0.12	0.75	Upland field	24	160	80	136
		A22-SL	2022	60	*tof11*	–	24-May	0.12	0.75	Upland field	24	160	80	136

*^a^* FU-RIL population was divided into two groups based on maturity, early maturity group (EMG) and late maturity group (LMG).

**Table 3.** Major QTLs for lodging angle in FU-RILs

Chr (LG)*^a^*	Year	Test name	Group*^b^*	Position (Mb)	Nearest marker	LOD*^c^*	Additive effect*^d^*	R^2^ (%)*^e^*	QTL name
13 (F)	2018	I18-FUE	EMG	37.9	BARCSOYSSR_13_1538	3.7	0.23	20.9	*qLT13-1*
		I18-FUL	LMG	37.9	BARCSOYSSR_13_1538	3.6	0.35	20.7	
19 (L)	2017	H17-FUL	LMG	43.0	Satt561	4.9	–0.24	16.4	*qLT19-1*
	2018	I18-FUL	LMG	43.0	Satt561	4.8	–0.29	14.4	

*^a^* Linkage groups. *^b^* RIL population was divided into two groups based on maturity, early maturity group (EMG) and late maturity group (LMG). *^c^* Logarithm of odds. *^d^* Additive effect of the FY allele on the QTL. *^e^* Phenotypic variance explained by the QTL.

**Table 4.** Relationship between the *qLT13-1* allele and other agronomic traits in FU-RILs

Year	Test name	Group*^a^*	Genotype at *qLT13-1* locus*^b^*	Lodging angle (°)	Days to flowering	Main stem length (cm)	Number of main stem nodes	100-seed weight (g)	Seed yield (g/plant)
2018	A18-FUE	EMG	FY	17.4	70.5	71.3	18.4	20.5	28.0
			UA	13.1	70.7	63.2	17.9	19.9	26.8
				ns	ns	***	*	ns	ns
2017	I17-FUE	EMG	FY	15.2	54.4	77.1	16.0	22.5	25.3
			UA	9.9	54.6	69.5	16.1	23.2	26.5
				**	ns	**	ns	ns	ns
2018	I18-FUE	EMG	FY	38.9	52.2	75.1	16.5	23.1	21.6
			UA	30.1	52.7	66.9	16.4	23.4	19.4
				**	ns	*	ns	ns	ns
2017	I17-FUL	LMG	FY	31.3	45.9	25.1	8.9	24.2	23.8
			UA	26.6	45.7	21.1	8.6	25.0	23.0
				*	ns	***	*	ns	ns
2018	I18-FUL	LMG	FY	30.5	47.3	84.8	17.5	22.8	21.4
			UA	16.3	47.4	71.6	16.9	23.8	22.3
				***	ns	***	ns	ns	ns
2017	H17-FUL	LMG	FY	26.9	ND*^c^*	64.1	15.9	20.7	21.7
			UA	19.6	ND	54.6	14.9	20.7	22.0
				*		***	***	ns	ns
2017	F17-FUL	LMG	FY	28.1	53.0	61.0	16.9	20.4	34.9
			UA	22.0	53.1	53.2	16.3	21.1	40.9
				*	ns	***	ns	ns	**

*, **, *** significantly different values at *p* < 0.05, *p* < 0.01, and *p* < 0.001, respectively, based on Welch’s *t*-test.; ns, not significant. *^a^* RIL population was divided into two groups based maturity, early (EMG) and late (LMG). *^b^* BARCSOYSSR_13_1538, the marker nearest to *qLT13-1* was used for genotyping. *^c^* No data.

**Table 5.** Relationship between the *qLT19-1* allele and other agronomic traits in FU-RILs

Year	Test name	Group*^a^*	Genotype at *qLT19-1* locus*^b^*	Lodging angle (°)	Days to flowering	Main stem length (cm)	Number of main stem nodes	100-seed weight (g)	Seed yield (g/plant)
2018	A18-FUE	EMG	FY	13.0	70.3	64.0	18.1	20.4	26.8
			UA	16.2	71.2	68.9	18.1	19.9	27.7
				ns	ns	*	ns	ns	ns
2017	I17-FUE	EMG	FY	11.3	54.1	71.6	16.1	23.3	25.2
			UA	12.7	55.0	73.8	16.0	22.7	27.1
				**	ns	ns	ns	ns	ns
2018	I18-FUE	EMG	FY	32.7	51.5	67.1	16.4	23.9	18.2
			UA	34.5	53.6	72.9	16.4	23.1	23.4
				ns	***	*	ns	ns	**
2017	I17-FUL	LMG	FY	27.5	45.8	73.9	16.8	25.9	23.5
			UA	31.9	45.9	78.8	16.5	23.3	23.1
				*	ns	ns	ns	***	ns
2018	I18-FUL	LMG	FY	19.3	47.1	78.2	17.5	24.5	21.8
			UA	31.2	47.8	81.3	17.0	22.7	21.8
				***	ns	ns	ns	***	ns
2017	H17-FUL	LMG	FY	18.4	ND*^c^*	58.2	15.3	21.6	22.2
			UA	30.1	ND	62.3	15.6	19.8	21.4
				**		ns	ns	**	ns
2017	F17-FUL	LMG	FY	22.7	52.3	57.3	16.9	21.8	36.2
			UA	28.7	54.0	59.0	16.5	19.6	38.1
				*	*	ns	ns	***	ns

*, **, *** significantly different values at *p* < 0.05, *p* < 0.01, and *p* < 0.001, respectively, based on Welch’s *t*-test.; ns, not significant. *^a^* RIL population was divided into two groups based maturity, early (EMG) and late (LMG). *^b^* Satt516, the marker nearest to *qLT19-1*, was used for genotyping. *^c^* No data.

**Table 6.** Relationship between *qLT13-1* allele and lodging related traits in the SL-RILs

Year	Test name	Genotype at *qLT13-1* locus*^a^*	Lodging score	Days to flowering	Main stem length (cm)	Number of main stem nodes	100-seed weight (g)	Seed yield (g/plant)
2019	A19-SL	SY	3.2	55.5	118.2	25.5	24.6	44.0
		LD	2.3	55.2	97.9	24.2	24.2	44.6
			**	ns	***	*	ns	ns
2020	A20-SL	SY	2.5	52.9	94.3	23.7	20.3	34.1
		LD	2.0	53.3	81.8	23.4	20.2	34.8
			ns	ns	**	ns	ns	ns
2021	A21-SL	SY	2.4	49.5	119.5	25.7	23.5	30.9
		LD	1.2	49.2	99.2	25.6	23.4	32.6
			***	ns	***	ns	ns	ns
2022	A22-SL	SY	2.5	55.4	110.7	26.2	19.4	29.0
		LD	1.5	55.3	90.6	24.8	20.0	26.6
			***	ns	***	**	ns	ns

*, **, *** significantly different values at *p* < 0.05, *p* < 0.01, and *p* < 0.001, respectively, based on Welch’s *t*-test.; ns, not significant. *^a^* BARCSOYSSR_13_1545 was used for genotyping.

## References

[B1] Ainsworth, E.A., C.R. Yendrek, J.A. Skoneczka and S.P. Long (2012) Accelerating yield potential in soybean: potential targets for biotechnological improvement. Plant Cell Environ 35: 38–52. 21689112 10.1111/j.1365-3040.2011.02378.x

[B2] Bernard, R.L. (1971) Two major genes for time of flowering and maturity in soybeans. Crop Sci 11: 242–244.

[B3] Bernard, R.L. (1972) Two genes affecting stem termination in soybeans. Crop Sci 12: 235–239.

[B4] Board, J. (2001) Reduced lodging for soybean in low plant population is related to light quality. Crop Sci 41: 379–384.

[B5] Buzzell, R.I. (1971) Inheritance of a soybean flowering response to fluorescent-daylength conditions. Can J Genet Cytol 13: 703–707.

[B6] Buzzell, R.I. and H.D. Voldeng (1980) Inheritance of insensitivity to long daylength. Soybean Genet Newsl 7: 26–29.

[B7] Chen, H. (2018) The spatial patterns in long-term temporal trends of three major crops’ yields in Japan. Plant Prod Sci 21: 177–185.

[B8] Chen, P., C.H. Sneller, J.C. Rupe, R.D. Riggs and R.T. Robbins (2006) Registration of ‘UA 4805’ soybean. Crop Sci 46: 974–974.

[B9] Cober, E.R. and M.J. Morrison (2010) Regulation of seed yield and agronomic characters by photoperiod sensitivity and growth habit genes in soybean. Theor Appl Genet 120: 1005–1012. 20012856 10.1007/s00122-009-1228-6

[B10] Cooper, R.L. (1971) Influence of soybean production practices on lodging and seed yield in highly productive environments. Agron J 63: 490–493.

[B11] de Felipe, M., J. Gerde and J. Rotundo (2016) Soybean genetic gain in maturity groups III to V in Argentina from 1980 to 2015. Crop Sci 56: 3066–3077.

[B12] Diers, B.W., T.R. Cary, D.J. Thomas and C.D. Nickell (2006) Registration of ‘LD00–3309’ soybean. Crop Sci 46: 1384.

[B13] Fujii, K., T. Sayama, K. Takagi, K. Kosuge, K. Okano, A. Kaga and M. Ishimoto (2018) Identification and dissection of single seed weight QTLs by analysis of seed yield components in soybean. Breed Sci 68: 177–187. 29875601 10.1270/jsbbs.17098 PMC5982185

[B14] Funatsuki, H., M. Hajika, T. Yamada, M. Suzuki, S. Hagihara, Y. Tanaka, S. Fujita, M. Ishimoto and K. Fujino (2012) Mapping and use of QTLs controlling pod dehiscence in soybean. Breed Sci 61: 554–558. 23136494 10.1270/jsbbs.61.554 PMC3406785

[B15] Funatsuki, H., M. Suzuki, A. Hirose, H. Inaba, T. Yamada, M. Hajika, K. Komatsu, T. Katayama, T. Sayama, M. Ishimoto et al. (2014) Molecular basis of a shattering resistance boosting global dissemination of soybean. Proc Natl Acad Sci USA 111: 17797–17802. 25468966 10.1073/pnas.1417282111 PMC4273335

[B16] Hwang, S. and T.G. Lee (2019) Integration of lodging resistance QTL in soybean. Sci Rep 9: 6540. 31024048 10.1038/s41598-019-42965-6 PMC6484036

[B17] Igita, K., K. Sasaki, S. Katayama and A. Okabe (1984) Yearly variability of agronomic characters and yield components of soybean in crop situation experiment. Tohoku Agricultural Research 35: 75–76 (in Japanase).

[B18] Jin, J., X. Liu, G. Wang, L. Mi, Z. Shen, X. Chen and S. Herbert (2010) Agronomic and physiological contributions to the yield improvement of soybean cultivars released from 1950 to 2006 in Northeast China. Field Crops Res 115: 116–123.

[B19] Kato, S., T. Sayama, M. Ishimoto, S. Yumoto, A. Kikuchi and T. Nishio (2018) The effect of stem growth habit on single seed weight and seed uniformity in soybean (*Glycine max* (L.) Merrill). Breed Sci 68: 352–359. 30100802 10.1270/jsbbs.17137 PMC6081300

[B20] Khosla, S., M. Augustus and V. Brahmachari (1999) Sex-specific organisation of middle repetitive DNA sequences in the mealybug *Planococcus lilacinus*. Nucleic Acids Res 27: 3745–3751. 10471745 10.1093/nar/27.18.3745 PMC148631

[B21] Kobayashi, S. and Y. Kunimitsu (2016) Change in soybean productivity and its trend in Japan: measurement by the Tornqvist index. Journal of Rural Economics 88: 173–177 (in Japanese with English summary).

[B22] Kosambi, D.D. (1943) The estimation of map distances from recombination values. Ann Eugen 12: 172–175.

[B23] Lander, E.S., P. Green, J. Abrahamson, A. Barlow, M.J. Daly, S.E. Lincoln and L.A. Newberg (1987) MAPMAKER: an interactive computer package for constructing primary genetic linkage maps of experimental and natural populations. Genomics 1: 174–181. 3692487 10.1016/0888-7543(87)90010-3

[B24] Lee, S.H., M.A. Bailey, M.A.R. Mian, E.R. Shipe, D.A. Ashley, W.A. Parrott, R.S. Hussey and H.R. Boerma (1996) Identification of quantitative trait loci for plant height, lodging, and maturity in a soybean population segregating for growth habit. Theor Appl Genet 92: 516–523. 24166318 10.1007/BF00224553

[B25] Lin, M.S. and R.L. Nelson (1988) Relationship between plant height and flowering date in determinate soybean. Crop Sci 28: 27–30.

[B26] Lu, S., L. Dong, C. Fang, S. Liu, L. Kong, Q. Cheng, L. Chen, T. Su, H. Nan, D. Zhang et al. (2020) Stepwise selection on homeologous *PRR* genes controlling flowering and maturity during soybean domestication. Nat Genet 52: 428–436. 32231277 10.1038/s41588-020-0604-7

[B27] Mansur, L.M., K.G. Lark, H. Kross and A. Oliveira (1993) Interval mapping of quantitative trait loci for reproductive, morphological, and seed traits of soybean (*Glycine max* L.). Theor Appl Genet 86: 907–913. 24193996 10.1007/BF00211040

[B28] Morrison, M., H. Voldeng and E. Cober (2000) Agronomic changes from 58 years of genetic improvement of short-season soybean cultivars in Canada. Agron J 92: 780–784.

[B29] Ohba, T., I. Iwata, C. Takezaki, N. Kudo, K. Igita, T. Shodai, M. Hara, M. Ikeda, S. Takayanagi, M. Shimotsu et al. (1982) A new soybean cultivar Fukuyutaka. Bulletin of the Kyushu National Agricultural Experiment Station 22: 405–432 (in Japanase with English summary).

[B30] Orf, J.H., K. Chase, T. Jarvik, L.M. Mansur, P.B. Cregan, F.R. Adler and K.G. Lark (1999) Genetics of soybean agronomic traits: I. Comparison of three related recombinant inbred populations. Crop Sci 39: 1642–1651.

[B31] Palomeque, L., L.J. Liu, W.B. Li, B.R. Hedges, E.R. Cober, M.P. Smid, L. Lukens and I. Rajcan (2010) Validation of mega-environment universal and specific QTL associated with seed yield and agronomic traits in soybeans. Theor Appl Genet 120: 997–1003. 20012262 10.1007/s00122-009-1227-7

[B32] Philbrook, B.D. and E.S. Oplinger (1989) Soybean field losses as influenced by harvest delays. Agron J 81: 251–258.

[B33] Qin, C., Y.H. Li, D. Li, X. Zhang, L. Kong, Y. Zhou, X. Lyu, R. Ji, X. Wei, Q. Cheng et al. (2023) *PH13* improves soybean shade traits and enhances yield for high-density planting at high latitudes. Nat Commun 14: 6813. 37884530 10.1038/s41467-023-42608-5 PMC10603158

[B34] Rehman, M., D. Luo, S. Mubeen, J. Pan, S. Cao, W. Saeed and P. Chen (2024) Progress in agronomic crops lodging resistance and prevention: A review. J Agron Crop Sci 210: e12785.

[B35] Rogers, J., P.Y. Chen, A.N. Shi, B. Zhang, A. Scaboo, S.F. Smith and A.L. Zeng (2015) Agronomic performance and genetic progress of selected historical soybean varieties in the southern USA. Plant Breed 134: 85–93.

[B36] Saitoh, K., K. Nishimura and T. Kitahara (2012) Effect of lodging on seed yield of field-grown soybean—Artificial lodging and lodging preventing treatments. Jpn J Crop Sci 81: 27–32 (in Japanese with English summary).

[B37] Sayama, T., T.Y. Hwang, K. Komatsu, Y. Takada, M. Takahashi, S. Kato, H. Sasama, A. Higashi, Y. Nakamoto, H. Funatsuki et al. (2011) Development and application of a whole-genome simple sequence repeat panel for high-throughput genotyping in soybean. DNA Res 18: 107–115. 21454301 10.1093/dnares/dsr003 PMC3077039

[B38] Shaw, R.H. and C.R. Weber (1967) Effects of canopy arrangements on light interception and yield of soybeans. Agron J 59: 155–159.

[B39] Song, Q.J., G.F. Jia, Y.L. Zhu, D. Grant, R.T. Nelson, E.Y. Hwang, D.L. Hyten and P.B. Cregan (2010) Abundance of SSR motifs and development of candidate polymorphic SSR markers (BARCSOYSSR_1.0) in soybean. Crop Sci 50: 1950–1960.

[B40] Takahashi, M., R. Matsunaga, K. Komatsu, Y. Nakazawa, M. Hajika, S. Sakai and K. Igita (2004) New soybean caltivar “Sachiyutaka”. Bulletin of the National Agricultural Research Center for Kyushu Okinawa Region 45: 15–39 (in Japanese with English summary).

[B41] Tsubokura, Y., S. Watanabe, Z.J. Xia, H. Kanamori, H. Yamagata, A. Kaga, Y. Katayose, J. Abe, M. Ishimoto and K. Harada (2014) Natural variation in the genes responsible for maturity loci *E1*, *E2*, *E3* and *E4* in soybean. Ann Bot 113: 429–441. 24284817 10.1093/aob/mct269 PMC3906962

[B42] Tsuchiya, T. (1987) Physiological and genetic analysis of pod shattering in soybeans. Jpn Agric Res Q 21: 166–175.

[B43] Tuinstra, M.R., G. Ejeta and P.B. Goldsbrough (1997) Heterogeneous inbred family (HIF) analysis: a method for developing near-isogenic lines that differ at quantitative trait loci. Theor Appl Genet 95: 1005–1011.

[B44] Uchikawa, O., M. Miyazaki and K. Tanaka (2006) The relationship lodging of soybean and the combine harvesting loss in Fukuoka Prefecture in 2004. Report of the Kyushu Branch of the Crop Science Society of Japan 72: 32–34 (in Japanese).

[B45] Umburanas, R., J. Kawakami, E. Ainsworth, J. Favarin, L. Anderle, D. Dourado-Neto and K. Reichardt (2022) Changes in soybean cultivars released over the past 50 years in southern Brazil. Sci Rep 12: 508. 35017557 10.1038/s41598-021-04043-8 PMC8752842

[B46] Wang, X., M.W. Li, F.L. Wong, C.Y. Luk, C.Y.L. Chung, W.S. Yung, Z.L. Wang, M. Xie, S.K. Song, G. Chung et al. (2021) Increased copy number of *gibberellin 2-oxidase 8* genes reduced trailing growth and shoot length during soybean domestication. Plant J 107: 1739–1755. 34245624 10.1111/tpj.15414

[B47] Watanabe, S., R. Hideshima, Z.J. Xia, Y. Tsubokura, S. Sato, Y. Nakamoto, N. Yamanaka, R. Takahashi, M. Ishimoto, T. Anai et al. (2009) Map-based cloning of the gene associated with the soybean maturity locus *E3*. Genetics 182: 1251–1262. 19474204 10.1534/genetics.108.098772 PMC2728863

[B48] Weber, C.R. and W.R. Fehr (1966) Seed yield losses from lodging and combine harvesting in soybeans. Agron J 58: 287–289.

[B49] Wilcox, J.R. (2001) Sixty years of improvement in publicly developed elite soybean lines. Crop Sci 41: 1711–1716.

[B50] Wilcox, J.R. and T. Sediyama (1981) Interrelationships among height, lodging and yield in determinate and indeterminate soybeans. Euphytica 30: 323–326.

[B51] Wu, T., S. Sun, C. Wang, W. Lu, B. Sun, X. Song, X. Han, T. Guo, W. Man, Y. Cheng et al. (2015) Characterizing changes from a century of genetic improvement of soybean cultivars in Northeast China. Crop Sci 55: 2056–2067.

[B52] Yamada, T., H. Funatsuki, A. Kaga, K. Takahashi, N. Yamada, K. Hirata, N. Oki, T. Sayama, M. Ishimoto and M. Hajika (2013) Production of new soybean lines by back-crossing and marker assisted selection for shattering resistance and maturity loci. Bulletin of the NARO Institute of Crop Science 14: 13–22 (in Japanese with English summary).

[B53] Yamada, T., M. Hajika, H. Funatsuki, K. Takahashi, K. Hirata, A. Hishinuma and J. Tanaka (2017) Causal analysis of yield-increase by introgression of shattering resistance gene *pdh1* in soybean. Jpn J Crop Sci 86: 251–257 (in Japanese with English summary).

[B54] Yamanaka, N., S. Watanabe, K. Toda, M. Hayashi, H. Fuchigami, R. Takahashi and K. Harada (2005) Fine mapping of the *FT1* locus for soybean flowering time using a residual heterozygous line derived from a recombinant inbred line. Theor Appl Genet 110: 634–639. 15657740 10.1007/s00122-004-1886-3

