# Supplementary material for: Genetic dissection of soybean lodging tolerance in recombinant inbred-line populations of major Japanese and modern US varieties
Source: Breed Sci. 2025 Jun 21;75(3):224–35. doi: 10.1270/jsbbs.24088 (PMC12457785; doi:10.1270/jsbbs.24088)
Supplement: Supplementary file 1 — Supplemental Figures [file 75_224_s1.pdf]

doi: 10.1270/jsbbs.24088

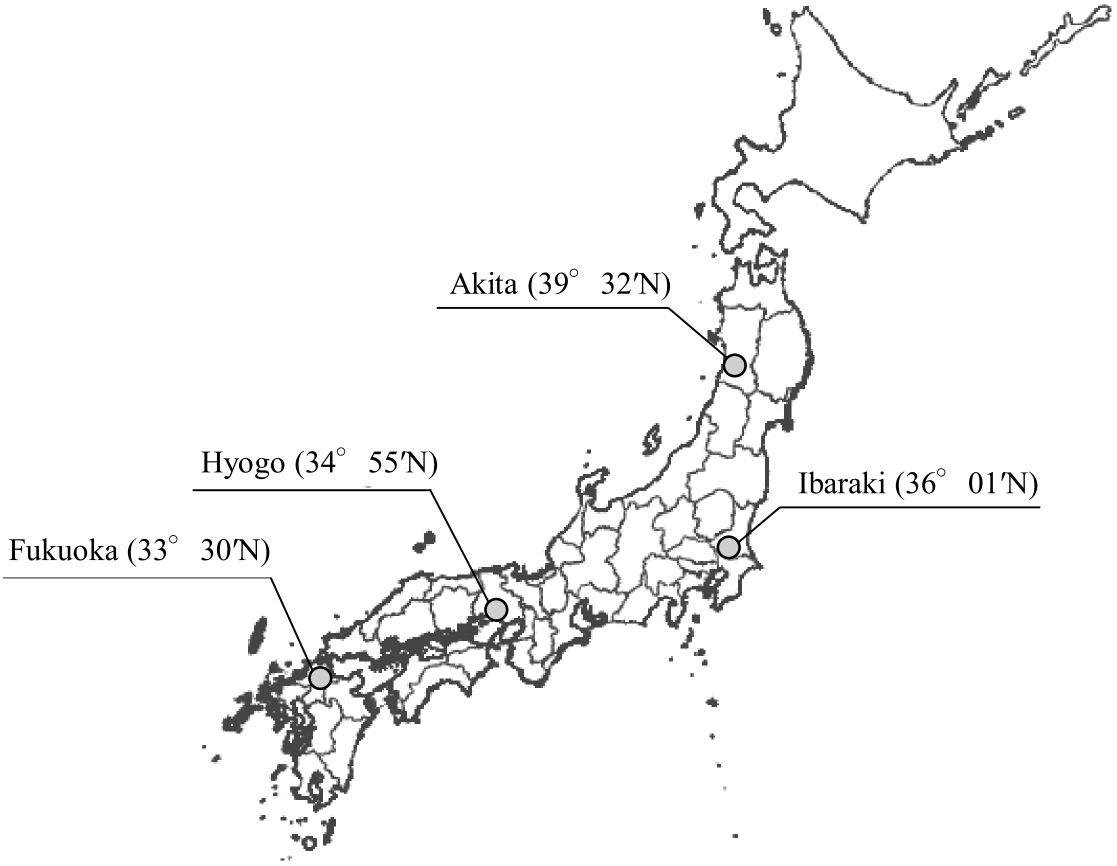

Supplemental Fig. 1 The circles on the map indicate the locations where each experiment was conducted. Latitude is shown after the place name.

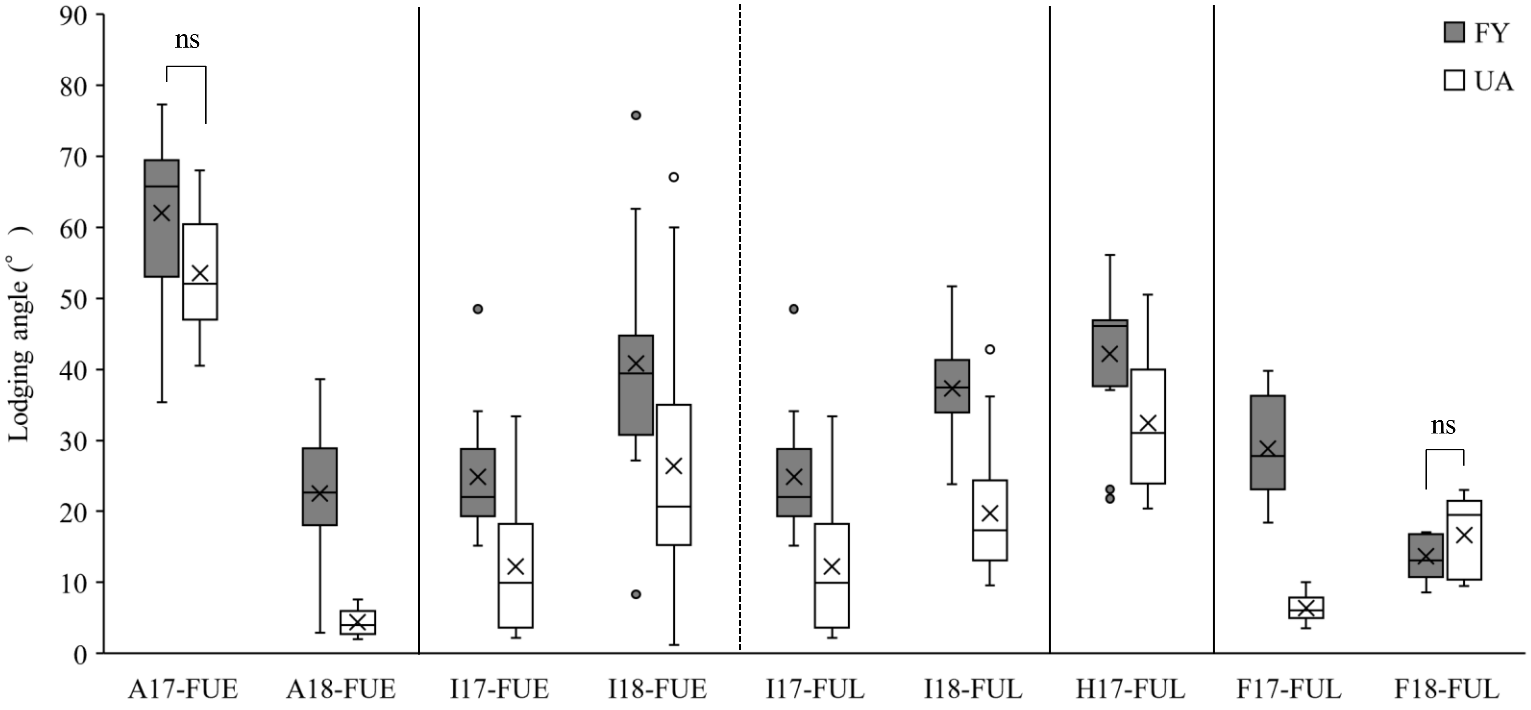

Supplemental Fig. 2 Comparisons of lodging angle between FY and UA. Significant differences ($p < 0.05$) were detected using Welch's *t*-test with log-transformed lodging angle values, except for the pairs which are shown as ns (not significant).

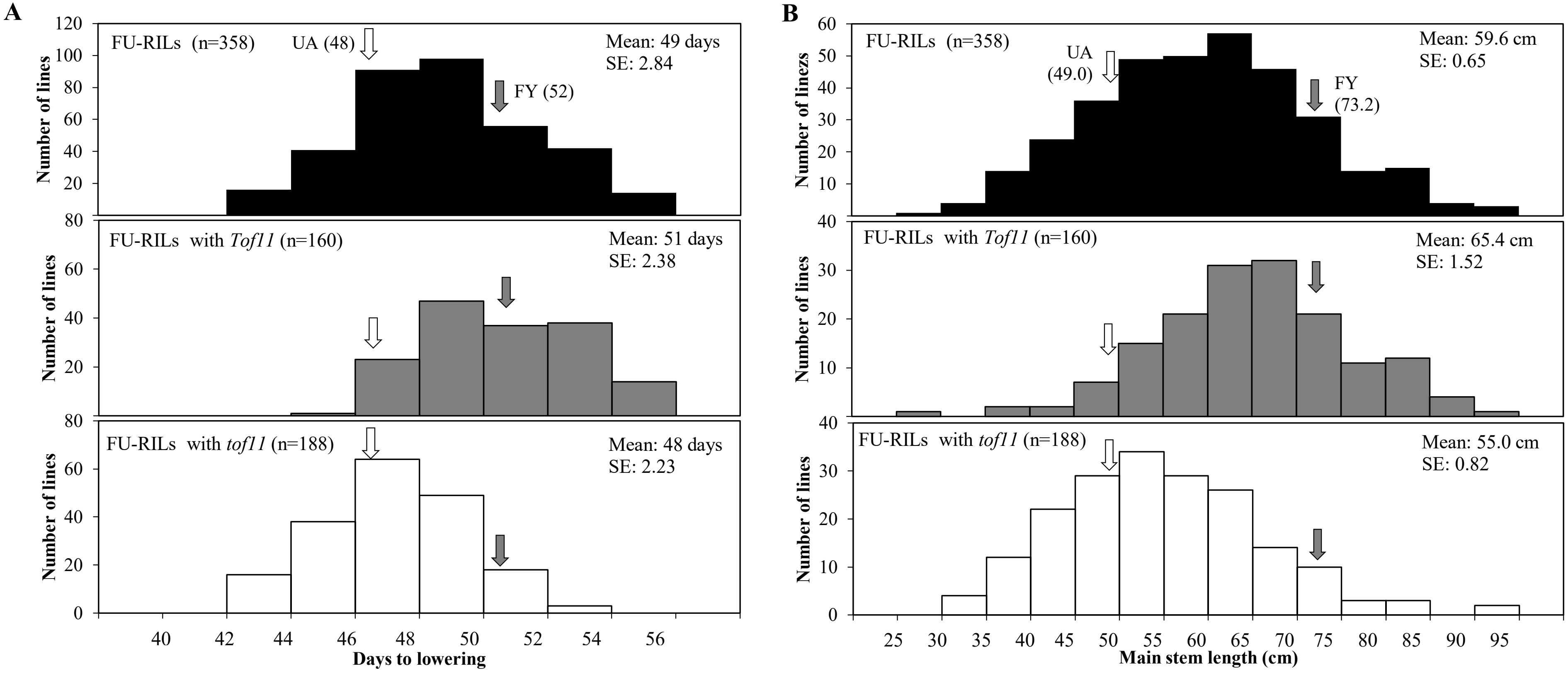

Supplemental Fig. 3 Frequency distribution of related to the *Tof11* genotype of FU-RIL. The population was sown in Ibaraki on July 24, 2016. (A) Frequency of distribution of days to flowering (FT). (B) Frequency of distribution of main stem length. In both panels, white and filled arrows indicate UA4805 (UA) and Fukuyutaka (FY), respectively, and the FT or main stem length of each is shown in parentheses. The actual number of RILs and the number of measurements differ due to missing values. The means and standard errors (SE) for each population are shown.

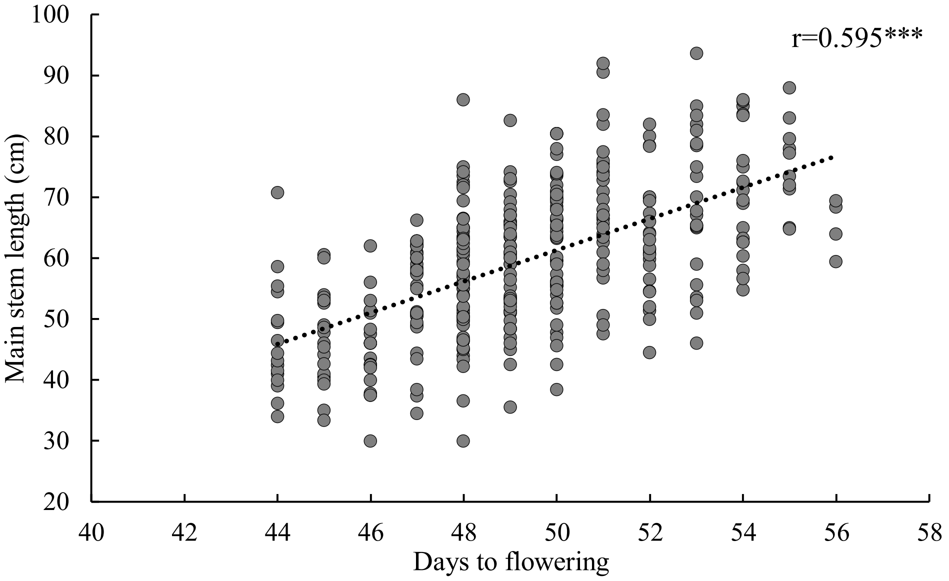

Supplemental Fig. 4 Relationships among main stem length and days to flowering (FT) of FU-RILs in Ibaraki in 2016. *** indicates significance at the 0.1% levels.

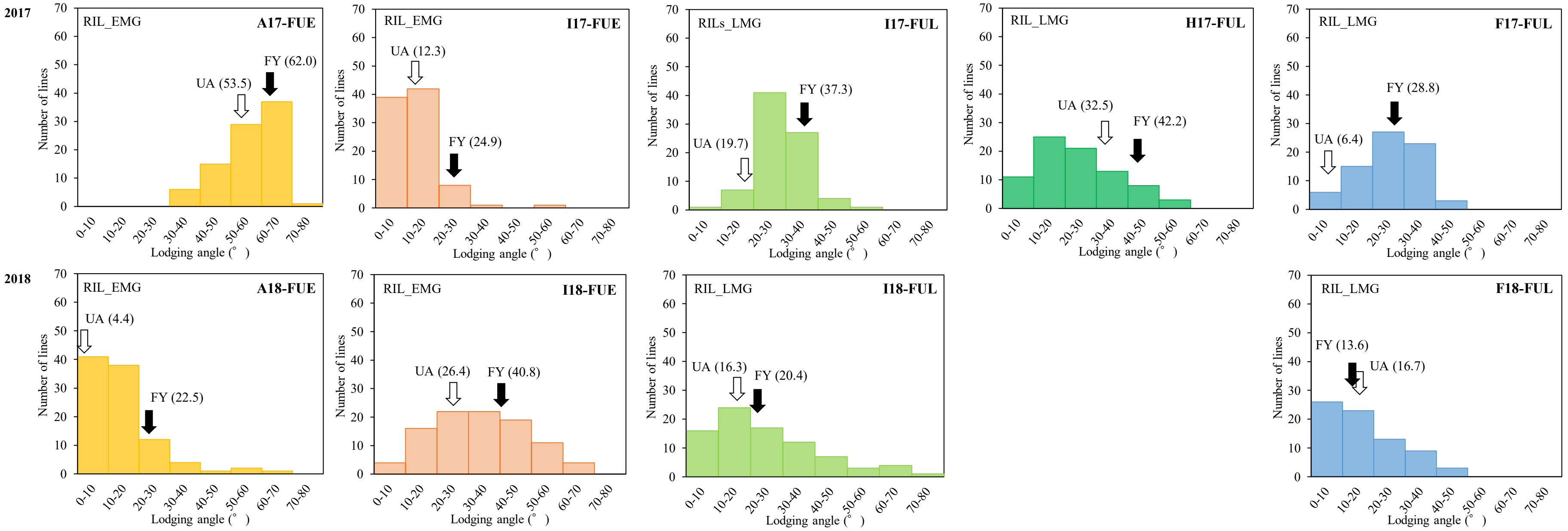

Supplemental Fig. 5 Frequency distribution related to the lodging angle. RILs were divided into two groups based on maturity: early maturity group (EMG) and late maturity group (LMG). White arrow and black arrow shows lodging angle of the parents, UA4805 (UA) and Fukuyutaka (FY), respectively.

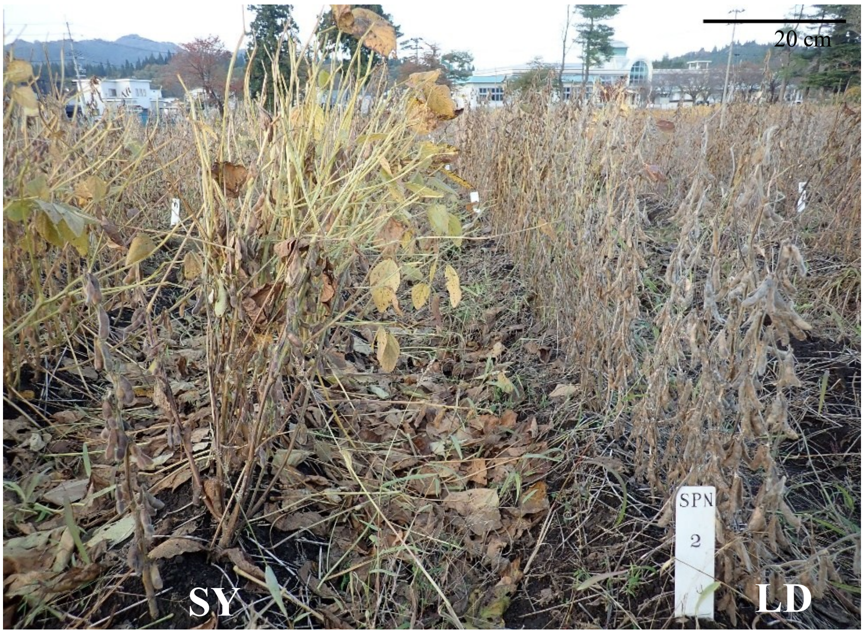

Supplemental Fig. 6 Representative plant images of Sachiyutaka (SY) and LD00-3309 (LD) in the field in Akita on 2 November 2023. They were sown on 30 May.

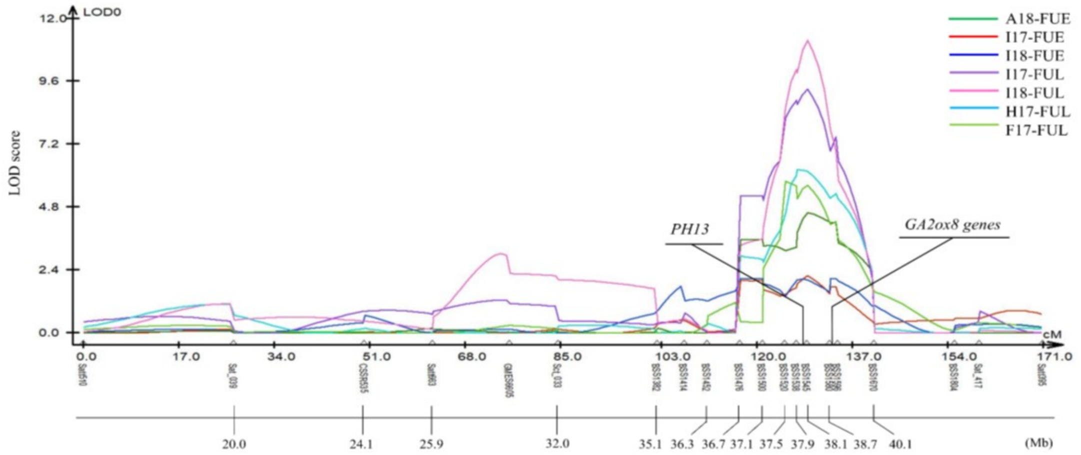

Supplemental Fig. 7 LOD plots of QTL associated main stem length result in using addional markers on Chr. 13. The marker name "BSS" stands for "BARCSOYSSR_13_." The region of *PH13* and copy number variation of *GA2ox8* genes are shown. The LOD threshold value at the 5% probability level was under 3.1 for significantly detected QTLs except for A18-FUE which was 3.6 (line not shown).

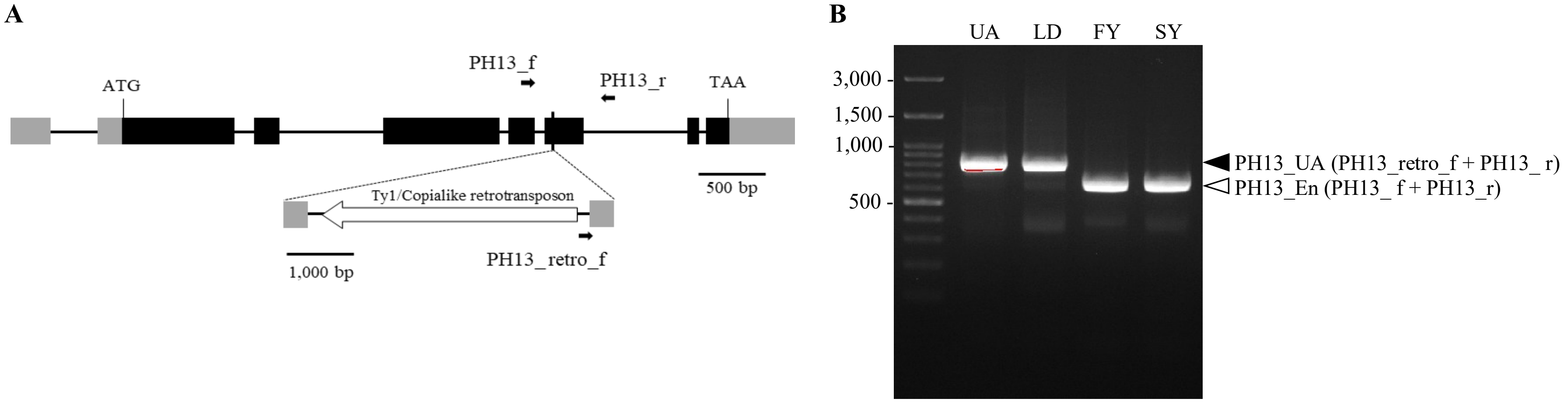

Supplemental Fig. 8 Confirmation of the insertion of a retrotransposon at *PH13* on Chr. 13. (A) Schematic representation of the *PH13* gene and the position of molecular markers to detect the presence of the retrotransposon. Solid and shaded boxes in the gene structure represent exons and untra nslated region , respectively. Filled arrows indicate marker position. Marker sequences were listed in Supplemental Table 1. (B) The presence of fragment insertions was c onfirmed by agarose gel electrophoresis. Bands indicated by filled arrows are specific to UA or LD; bands indicated by white arrows are specific to FY or SY.
