# Supplementary material for: Genetic dissection of soybean lodging tolerance in recombinant inbred-line populations of major Japanese and modern US varieties
Source: Breed Sci. 2025 Jun 21;75(3):224–35. doi: 10.1270/jsbbs.24088 (PMC12457785; doi:10.1270/jsbbs.24088)
Supplement: Supplementary file 2 — Supplemental Tables [file 75_224_s2.pdf]

Supplemental Table 1. DNA markers used for QTL analysis and/or QTL mapping on chromosome 13 and genotyping of target gene.

Marker	Primer sequence 1 (5'-3')	Primer sequence 2 (5'-3')	Start position on Chromosome 13	Objective	Reference
Sat_039	CAAGAATAATCTAAAGGTACACTT	AGTTAAAAAACCCACACAAC	19,958,853	QTL analysis	Song *et al.* 2010
CSSR535	AATCCTTCCAGTTTGGCAGA	TTTTGCAAATAGCTCCACCA	24,077,629	QTL analysis	Song *et al.* 2010
Satt663	GCGTCATGCAATGTTGTATAAT	GCGACTGCAGATAACTTGACTGGTAGT	25,936,876	QTL analysis	Song *et al.* 2010
Satt510	GCGAGTTTCGCCGTTACCACCTCAGCTT	CCCTCTTATTTCACCCTAAGACCTACAA	31,802,559	QTL analysis	Song *et al.* 2010
Sct_033	CTTTTAAATTATAATAGCATGATCT	TGCTAATTTAGATTACGTTATGT	31,951,960	QTL analysis	Song *et al.* 2010
GMES6605	AACCCCACTTCTTACCACCC	TGGCAGCATCGTTGTTGTAG	34,643,206	QTL analysis	Song *et al.* 2010
BARCSOYSSR_13_1382	CCTTCCACAACGGCTACAAT	GTGCTTGCAGGTTGTTGAGA	35,063,245	QTL mapping	Song *et al.* 2010
BARCSOYSSR_13_1414	GATGAACCAACTGGAGAGGC	TGACCAAAATATCAGCAGAAAAA	35,580,522	QTL mapping	Song *et al.* 2010
BARCSOYSSR_13_1444	CCCCGATCATTCACACATTT	GCCATTTGTGAGCAACTTAGG	36,219,682	QTL mapping	Song *et al.* 2010
BARCSOYSSR_13_1452	CATTGAGGGTCCTCATGCTT	AAAGTGAAAATTATCCAATTGTTTG	36,341,436	QTL mapping	Song *et al.* 2010
BARCSOYSSR_13_1476	CCTTTGTAAAGCTCAATGCAA	CGATAACGACCGTGATGAAT	36,721,574	QTL mapping	Song *et al.* 2010
BARCSOYSSR_13_1500	TGAAGTGTGGAATATCGACCA	CCTGAAAAGTTGATGGAAAATTG	37,062,997	QTL mapping	Song *et al.* 2010
BARCSOYSSR_13_1515	CTTCGGCCCACATAATCAAT	TCAGTCAACAAAAAGATTTGGAA	37,389,209	QTL mapping	Song *et al.* 2010
BARCSOYSSR_13_1520	TGGTGAAATAACTTCAGGCGA	CATGTACGCACGCATACAAAT	37,468,670	QTL mapping	Song *et al.* 2010
BARCSOYSSR_13_1538	AACTGCAGTCAACCATGCTG	TTGTCCCTGAAGACCTCCAC	37,938,252	QTL analysis / mapping	Song *et al.* 2010
BARCSOYSSR_13_1540	AACCTGCATTCGTTCCTGTC	GGGTAGTTGAGATAAGAGGATGGA	37,962,104	QTL mapping	Song *et al.* 2010
BARCSOYSSR_13_1545	GGTTTCTTTTTATTTTGTCAGGGA	AACTTTGACCAGAAAGTAATTCAAAA	38,128,643	QTL mapping	Song *et al.* 2010
BARCSOYSSR_13_1580	TGATTGAAACCCAATTTTCTCC	GAGAGGCCCCTTCTTAGCAT	38,691,963	QTL mapping	Song *et al.* 2010
BARCSOYSSR_13_1596	GGCTCCCATCTGTTCATCAT	GCGTGTGACTGAAGTTGGTG	38,927,530	QTL mapping	Song *et al.* 2010
BARCSOYSSR_13_1670	CTCCCCAATTTTATGCTCCA	GGAGATAACCACCACTTATTTTTG	40,113,638	QTL analysis / mapping	Song *et al.* 2010
BARCSOYSSR_13_1804	TGGTTCACGCTTGATGTTCT	CCAGATGTGATATGATGTTTTAGC	42,704,014	QTL analysis	Song *et al.* 2010
WGSP13_0160	GCGAATATGGCGTTGAAAATAGTGAT	GCGACCCAGATTCTGTGCTAAGA	43,308,000	QTL mapping	Fujii *et al.* 2018
Satt395	CGCGCTAGTTGAATGAATGT	GCGCATTGAGGAATTTTTTAT	45,320,320	QTL analysis	Song et al. 2010
PH13_f	ACGGTTCTGTCAAGCTATGGAG		37,815,256	Genotyping of *PH13*	This study
PH13_ retro_f	AAGGGTTCAATCCTTAGTGACACCCTGAT		-	Genotyping of *PH13*	This study
PH13_r		CAAGTGAACTGGCTTTGTTTTTTTGGTGG	37,814,613	Genotyping of *PH13*	This study
El _*Taq* αI/*Hin* fI	TCAGATGAAAGGGAGCAGTGTCAAAAGAAGT	TCCGATCTCATCACCTTTCC	-	Genotyping of *E1*	Tsubokura *et al.* 2014
E2_DraI	GCCCATCAGAGGCATGTCTTATT	GAGGCAGAGCCAAAGCCTAT	-	Genotyping of *E2*	Tsubokura *et al.* 2014
E2_InDel	TGTTGATATTACATGCACATGCAT	GGCAGTTTCACCTTCTTAGC	-	Genotyping of *E2*	Tsubokura *et al.* 2014
E3E4_Mix	TGGGTCTTCAGTTCAGTTGG	CTAAGTCCGCCTCTGGTTTCAG	-	Genotyping of *E3* and *E4*	Tsubokura *et al.* 2014
		CGGTCAAGAGCCAACATGAG	-	Genotyping of *E3* and *E4*	Tsubokura *et al.* 2014
		GTCCTATACAATTCTTTACGACG	-	Genotyping of *E3* and *E4*	Tsubokura *et al.* 2014
	AGACGTAGTGCTAGGGCTAT	GCATCTCGCATCACCAGATCA	-	Genotyping of *E3* and *E4*	Tsubokura *et al.* 2014
		GCTCATCCCTTCGAATTCAG	-	Genotyping of *E3* and *E4*	Tsubokura *et al.* 2014

Chromosome positions refer to the 'Phytozome 13' assembly version v2.0.

Supplemental Table 2. Experiment conditions of the RHLs.

Location	Year	Group[a]	Planting Date	Plant separation distance (m)	Row spacing (m)	Field type	Fertilization (kg ha^{-1})			
							N	P_2O_5	K_2O	MgO
Ibaraki	2018	EMG	7-Jun	0.10	0.70	Upland field	30	500	100	300
	2019	EMG	6-Jun	0.10	0.70	Upland field	30	500	100	300
	2021	EMG	2-Jun	0.10	0.70	Upland field	30	500	100	300
	2018	LMG	3-Jul	0.10	0.70	Upland field	30	500	100	300
	2019	LMG	2-Jul	0.10	0.70	Upland field	30	500	100	300
	2021	LMG	30-Jun	0.10	0.70	Upland field	30	500	100	300

[a] Four RHLs were divided into two groups based on maturity: early (EMG) and late (LMG).

Supplemental Table 3. Heritability of lodging angle in Ibaraki.

Year	Group[a]		Lodging angle[b]	Main stem length
2017	EMG	*Ve*[c]	0.03	17.29
		Vg[d]	0.37	116.61
		H^2[e]	0.92	0.87
	LMG	*Ve*	0.04	6.21
		Vg	0.07	139.92
		H^2	0.65	0.96
2018	EMG	*Ve*	0.08	18.48
		Vg	0.17	179.93
		H^2	0.67	0.91
	LMG	*Ve*	0.14	16.90
		Vg	0.44	119.18
		H^2	0.76	0.88

[a] RIL population was devided into two groups based on maturuty, early (EMG) and late (LMG).

[b] Lodging angle data was logarithmically transformed.

[c] Variance attributable to environment.

[d] Variance attributable to genotype.

[e] Broad-sense heritability.

Supplemental Table 4. Pairwise correlation coefficients of the lodging angle of RILs, comparing among 2 years and 4 locations.

Group of the RILs[a]			EMG				LMG				
	Year		2017		2018		2017			2018	
		Test name[b]	A17-FUE	I17-FUE	A18-FUE	I18-FUE	I17-FUL	H17-FUL	F17-FUL	I18-FUL	F18-FUL
EMG	2017	A17-FUE		-0.04^{ns}	0.17^{ns}	-0.02^{ns}	-	-	-	-	-
		I17-FUE	-0.04^{ns}		0.42***	0.50***	-	-	-	-	-
	2018	A18-FUE	0.17^{ns}	0.42***		0.36***	-	-	-	-	-
		I18-FUE	-0.02^{ns}	0.50***	0.36***		-	-	-	-	-
LMG	2017	I17-FUL	-	-	-	-		0.55***	0.43***	0.47***	0.23^{ns}
		H17-FUL	-	-	-	-	0.55***		0.43***	0.65***	0.39**
		F17-FUL	-	-	-	-	0.43***	0.43***		0.45***	0.31**
	2018	I18-FUL	-	-	-	-	0.47***	0.65***	0.45***		0.33*
		F18-FUL	-	-	-	-	0.23^{ns}	0.39**	0.31**	0.33*	

Lodging angle data is logarithmically transformed for statistical analysis.

*, **, *** significantly different at $p < 0.05$, $p < 0.01$, and $p < 0.001$, respectively, based on Welch's *t*-test; ns, not significant.

[a] RIL population was divided into two groups based on maturity, early (EMG) and late (LMG).

[b] Data shown in white were excluded from QTL analysis and subsequent experiments.

Supplemental Table 5. Comparison of the effect on lodging angle between RHLs and marker genotypes around *qLT13-1* .

									← Candidate *qLT13-1* region							→			
			(Mb)		35.1			36.3	36.7		37.4		37.9		38.1	38.7	43.3		
			Markers															Lodging angle[b] (°)	
Group[a]	RHL name	Fixed pairs	Sct_033	BARCSOYSSR_13_1382	BARCSOYSSR_13_1414	BARCSOYSSR_13_1444	BARCSOYSSR_13_1452	BARCSOYSSR_13_1476	BARCSOYSSR_13_1500	BARCSOYSSR_13_1515	BARCSOYSSR_13_1520	BARCSOYSSR_13_1538	BARCSOYSSR_13_1540	BARCSOYSSR_13_1545	BARCSOYSSR_13_1580	BARCSOYSSR_13_1670	WGSP13_0160	2018 or 2019[c]	2021
	FY	-	F	F	F	F	F	F	F	F	F	F	F	F	F	F	F		
	UA	-	U	U	U	U	U	U	U	U	U	U	U	U	U	U	U		
EMG	RHL-185	185-F	F	F	F	F	F	F	F	F	F	F	F	F	F	F	F	48.9	38.5
		185-U	F	F	F	F	F	F	U	U	U	U	U	U	U	U	F	36.1	17.8
																		*	*
EMG	RHL-186	186-F	F	F	F	F	F	F	F	F	F	F	F	U	U	U	U	21.0	51.7
		186-U	F	F	F	F	F	F	F	U	U	U	U	U	U	U	U	13.3	33.4
																		ns	**
LMG	RHL-026	026-F	U	U	U	U	U	U	F	F	F	F	F	F	F	F	F	26.5	15.9
		026-U	U	U	U	U	U	U	U	U	U	U	U	U	U	U	F	27.6	11.7
																		ns	*
LMG	RHL-277	277-F	U	U	U	U	U	U	U	F	F	F	F	F	F	F	F	25.4	16.9
		277-U	U	U	U	U	U	U	U	U	U	U	U	F	F	F	F	22.6	16.1
																		ns	ns

Each genotype of the marker was determined as FY allele (F) or UA allele (U).

*, ** significantly different at $p < 0.05$ and $p < 0.01$, respectively, based on Welch's t-test; ns, not significant.

[a] RIL population was devided into two groups based on maturuty, early (EMG) and late (LMG).

[b] Logarithmic-transformed data were used for the statistical analysis.

[c] 185-F and 185-U were evaluated in 2018; the remaining lines were evaluated in 2019.

[d] No data.
